# Influence of nutrition on stage-stratified survival in gastric cancer patients with postoperative complications

**DOI:** 10.18632/oncotarget.28179

**Published:** 2022-01-21

**Authors:** Noriyuki Hirahara, Takeshi Matsubara, Shunsuke Kaji, Yuki Uchida, Ryoji Hyakudomi, Tetsu Yamamoto, Kiyoe Takai, Yohei Sasaki, Koki Kawakami, Yoshitsugu Tajima

**Affiliations:** ^1^Department of Digestive and General Surgery, Shimane University Faculty of Medicine, Izumo, Shimane, Japan; ^2^Department of Surgery, Matsue Red Cross Hospital, Shimane, Matsue, Horomachi, Japan; ^3^Department of Surgery, Masuda Red Cross Hospital, Shimane, Masuda, Otoyoshi-cho, Japan

**Keywords:** gastric cancer, prognostic nutritional index, laparoscopic gastrectomy, postoperative complications, prognosis

## Abstract

Background: We assessed the relationship between preoperative prognostic nutritional index (PNI) and short- and long-term outcomes among gastric cancer patients because the clinical significance of PNI in these patients remains controversial.

Materials and Methods: We retrospectively reviewed the medical records of 434 consecutive patients who underwent curative laparoscopic gastrectomy for gastric cancer.

Results: Patients with postoperative complications had a significantly poorer overall survival (OS) than those without. On multivariate analyses, postoperative complications were independently associated with PNI value and operative procedure type. In the low PNI group (*n* = 118), those with postoperative complications experienced significantly poorer OS than those without complications. Among the low PNI group with pTNM stage I and II disease, those with postoperative complications experienced significantly worse OS than those without complications. However, among the high PNI group and patients with stage II and III disease in the low PNI group, OS was similar with respect to postoperative complications.

Conclusions: The present study confirmed that long-term prognosis was unaffected by postoperative complications in well-nourished gastric cancer patients. In addition, preoperative nutritional status and postoperative complications, may be crucial in determining the prognosis of gastric cancer, especially in early-stage cancer.

## INTRODUCTION

Cancer-associated malnutrition is a common and significant clinical issue because it is often unrecognized and undertreated. Gastric cancer patients are often malnourished at the time of diagnosis, which is related to impaired digestion, malabsorption, and bleeding and/or protein loss from the primary lesion [1, 2]. Recent studies have indicated that the preoperative nutritional and immunological statuses are not only associated with postoperative complications but also with long-term prognosis among cancer patients [3–5]. Moreover, several studies have demonstrated that postoperative complications adversely affect long-term survival [6–8]. Therefore, the preoperative assessment of nutritional status and preoperative nutritional management may help improve short-term outcome and long-term prognosis in gastric cancer patients who undergoing laparoscopic gastrectomy.

The prognostic nutrition index (PNI), which predicts the risk of postoperative complications, is calculated using serum albumin level and total number of lymphocytes [9]. Serum albumin has been reported to reflect nutrition and the immune response, being indicative of macrophage activation, tumor progression, and prognosis [10, 11]. Lymphocytes have been found to activate the adaptive immune system to stop cancer dispersion [12, 13]. Albumin level and lymphocyte count balance is closely linked to immune and nutritional status and is reported to affect the prognosis of cancer patients [10–13]. In addition, basic nutrition and systemic inflammation are also reported to be associated with the long-term prognosis of cancer patients [14–16]. However, the clinical significance of PNI in patients with gastric cancer remains controversial. Therefore, this study aimed to evaluate the relationship between preoperative nutrition and immunological status (using PNI) and short-term outcome and long-term prognosis, especially among stage-stratified gastric cancer patients undergoing curative gastrectomy.

## RESULTS

### Background characteristics and PNI

Based on a PNI cut-off of 44.2, 118 (27.2%) and 316 (72.8%), patients were classified into the low and high PNI groups, respectively. Significant differences were observed in age, body mass index (BMI), tumor diameter, tumor stage, pathological TNM (pTNM) stage, surgical procedure, intraoperative blood loss, and C-reactive protein (CRP) level (Table 1).

**Table 1:** Relationships between PNI and clinicopathological features

Characteristics	No. of Patients	PNI		*p* value
		<44.2 (*n* = 118)	≥44.2 (*n* = 316)	
Age (years)		77 (46–91)	69 (36–89)	<0.001
Sex				0.885
Male	303	83	220	
Female	131	35	96	
BMI		21.3 (14.0–30.5)	22.6 (16.5–40.4)	<0.001
Tumor location				0.315
EGJ	11	2	9	
U	85	28	57	
M	184	43	141	
L	154	45	109	
Tumor size (mm)		55 (7–170)	38 (3–180)	<0.001
Histological differentiation				0.151
Well	81	15	66	
Moderate	162	47	115	
Poor	191	56	135	
Depth of tumor				<0.001
T1a–1b	232	45	187	
2	56	14	42	
3	58	19	39	
4a–4b	88	40	48	
Lymph node metastasis				0.072
N0	284	66	218	
N1	48	15	33	
N2	53	20	33	
N3	49	17	32	
pTNM stage				<0.001
1a–1b	260	52	208	
2a–2b	69	24	45	
3a–3c	105	42	63	
Operative procedure				0.047
Total	91	34	57	
Proximal	44	10	34	
Distal	299	74	225	
Operation time (min)		394 (177–911)	384 (70–881)	0.825
Intraoperative blood loss		57.5 (0–2620)	30 (0–4070)	0.007
Postoperative complications				0.146
Present	128	41	87	
Absent	306	77	229	
CRP (mg/dl)		0.20 (0.01–11.10)	0.06 (0.002–6.31)	<0.001
CEA (ng/ml)		3.6 (0.8–163.3)	3.2 (0.7–161.1)	0.089
Adjuvant chemotherapy				0.143
Yes	124	38	86	
No	310	80	230	

### Background characteristics and postoperative complications

Among the 434 patients, 128 (29.4%) experienced postoperative complications (Table 2). Significant differences were observed in age, sex, tumor location, surgical procedure, operation time, and serum CRP level. The postoperative complications were not associated with PNI.

**Table 2:** Relationships between postoperative complications and clinicopathological features

Characteristics	No. of Patients	Postoperative complications		*p* value
		Absent (*n* = 306)	Present (*n* = 128)	
Age (years)		77 (46–91)	69 (36–89)	<0.001
Sex				0.025
Male	303	204	99	
Female	131	102	29	
BMI		22.2 (14.7–40.4)	22.3 (14.0–32.5)	0.354
Tumor location				<0.001
EGJ	11	5	6	
U	85	43	42	
M	184	142	42	
L	154	116	38	
Tumor size (mm)		40 (3–176)	40 (3–180)	0.654
Histological differentiation				0.851
Well	81	55	26	
Moderate	162	115	47	
Poor	191	136	55	
Depth of tumor				0.334
T1a-1b	232	172	60	
2	56	38	18	
3	58	37	21	
4a-4b	88	59	29	
Lymph node metastasis				0.337
N0	284	205	79	
N1	48	35	13	
N2	53	37	16	
N3	49	29	20	
pTNM stage				0.190
1a-1b	260	191	69	
2a-2b	69	48	21	
3a-3c	105	67	38	
Operative procedure				<0.001
Total	91	49	42	
Proximal	44	22	22	
Distal	299	235	64	
Operation time (min)		379 (70–911)	413 (207–836)	0.009
Intraoperative blood loss		40 (0–4070)	50 (0–1580)	0.200
CRP (mg/dl)		0.07 (0.01–6.31)	0.10 (0.002–11.10)	0.006
CEA (ng/ml)		3.2 (0.7–106.0)	3.4 (0.7–163.3)	0.199
PNI				0.146
≥44.2	316	229	87	
<44.2	118	77	41	
Adjuvant chemotherapy				0.894
Yes	124	88	36	
No	310	218	92	

### Impact of PNI on OS among all patients

Univariate analysis of risk factors for OS age, BMI, tumor diameter, tumor differentiation, pTNM stage, carcinoembryonic antigen (CEA), CRP level, PNI, postoperative complications, and postoperative adjuvant chemotherapy were significant relevant factors. In multivariate analyses, pTNM stage (*p* < 0.001), CEA (*p* = 0.003), and PNI (*p* < 0.001) were found to be independent predictive factors for OS (Table 3).

**Table 3:** Univariate and multivariate analyses of clinicopathological factors for overall survival

Variables	Category or characteristics	Patients (*n* = 368)	Univariate			Multivariate		
			HR	95% CI	*p* value	HR	95% CI	*p* value
Age	(<70/≥70)	166/202	1.617	1.043–2.505	0.032	1.068	0.659–1.731	0.790
Sex	(Female/Male)	114/254	1.604	0.974–2.642	0.063			
BMI	(≥18.5/<18.5)	332/36	1.905	1.075–3.375	0.027	1.396	0.740–2.634	0.304
Tumor size	(<5/≥5)	217/151	2.334	1.525–3.573	<0.001	0.912	0.523–1.589	0.745
Histological differentiation	(well & mod/poor)	163/205	1.656	1.087–2.522	0.019	1.473	0.930–2.331	0.099
pTNM stage	(1, 2/3)	282/86	4.000	2.612–6.125	<0.001	3.047	1.687–5.505	<0.001
CEA	(<5.0/≥5.0)	286/82	2.350	1.528–3.612	<0.001	1.975	1.255–3.107	0.003
CRP	(≦0.5/>0.5)	312/56	2.674	1.691–4.229	<0.001	1.249	0.709–2.201	0.441
PNI	(≥44.2/<44.2)	254/114	3.750	2.463–5.711	<0.001	2.808	1.701–4.637	<0.001
Postoperative Complications	(Absent/Present)	265/103	1.876	1.220–2.884	0.004	1.427	0.884–2.305	0.146
Adjuvant chemotherapy	(no/yes)	268/100	1.960	1.284–2.990	0.002	0.995	0.580–1.706	0.986

### OS stratified according to PNI

**Figure 1:**
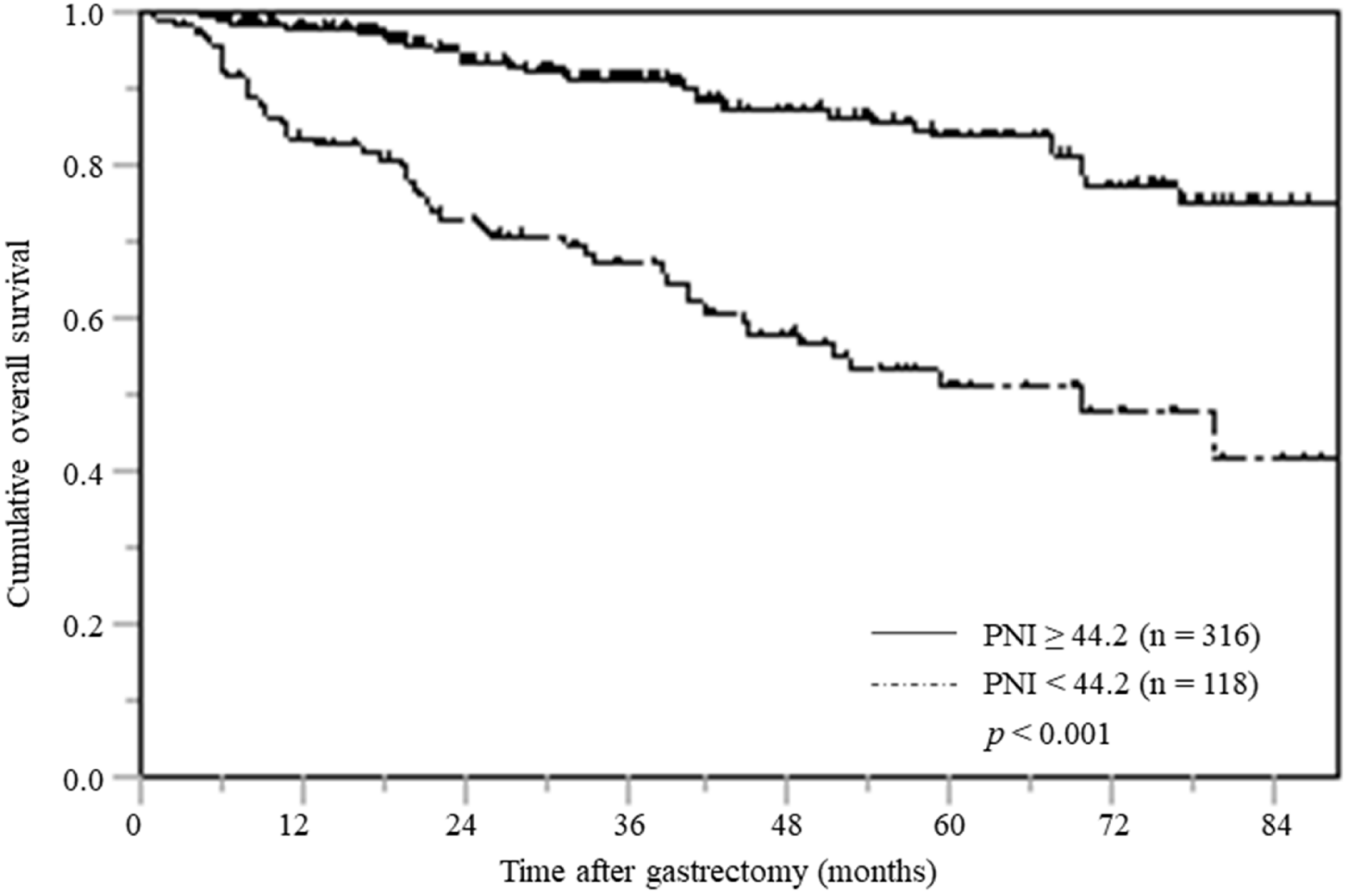
Overall survival based on PNI.

The OS curve for the low PNI group was significantly inferior to that of the high PNI group; this was reflected in the significantly lower OS rate at five-years after surgery (51.1% vs. 91.2%, respectively; *p* < 0.001) (Figure 1).

### Impact on postoperative complications

Univariate analysis of risk factors for postoperative complications showed that sex, CRP level, PNI, and surgical procedures were significant relevant factors. Multivariate analyses showed that PNI (*p* = 0.031) and surgical procedure (*p* = 0.011) were independent risk factors (Table 4).

**Table 4:** Univariate and multivariate analyses to assess the risk factors for postoperative complications

Variables	Category or characteristics	Patients (*n* = 368)	Univariate			Multivariate		
			OR	95% CI	*p* value	OR	95% CI	*p* value
Age	(<70/≥70)	166/202	1.452	0.955–2.207	0.081			
Sex	(Female/Male)	114/254	1.707	1.059–2.751	0.028	1.464	0.879–2.442	0.143
BMI	(≥18.5/<18.5)	332/ 36	0.933	0.450–1.937	0.853			
Tumor size	(<5/≥5)	217/151	0.972	0.638–1.481	0.895			
Histological differentiation	(well & mod/poor)	163/205	0.972	0.642–1.473	0.894			
pTNM stage	(1,2/3)	282/86	1.631	0.996–2.672	0.052			
CEA	(<5.0/≥5.0)	286/82	1.369	0.851–2.200	0.195			
CRP	(≦0.5/>0.5)	312/56	2.370	1.382–4.066	0.002	1.716	0.907–3.249	0.097
PNI	(≥44.2/<44.2)	254/114	6.228	2.147–18.066	<0.001	4.003	1.132–14.153	0.031
Operative Procedure	(proximal & distal/total)	286/82	2.300	1.389 – 3.806	0.001	1.968	1.167–3.319	0.011
Adjuvant chemotherapy	(no/yes)	268/100	0.969	0.613–1.532	0.894			

### Impact of postoperative complications on OS

**Figure 2:**
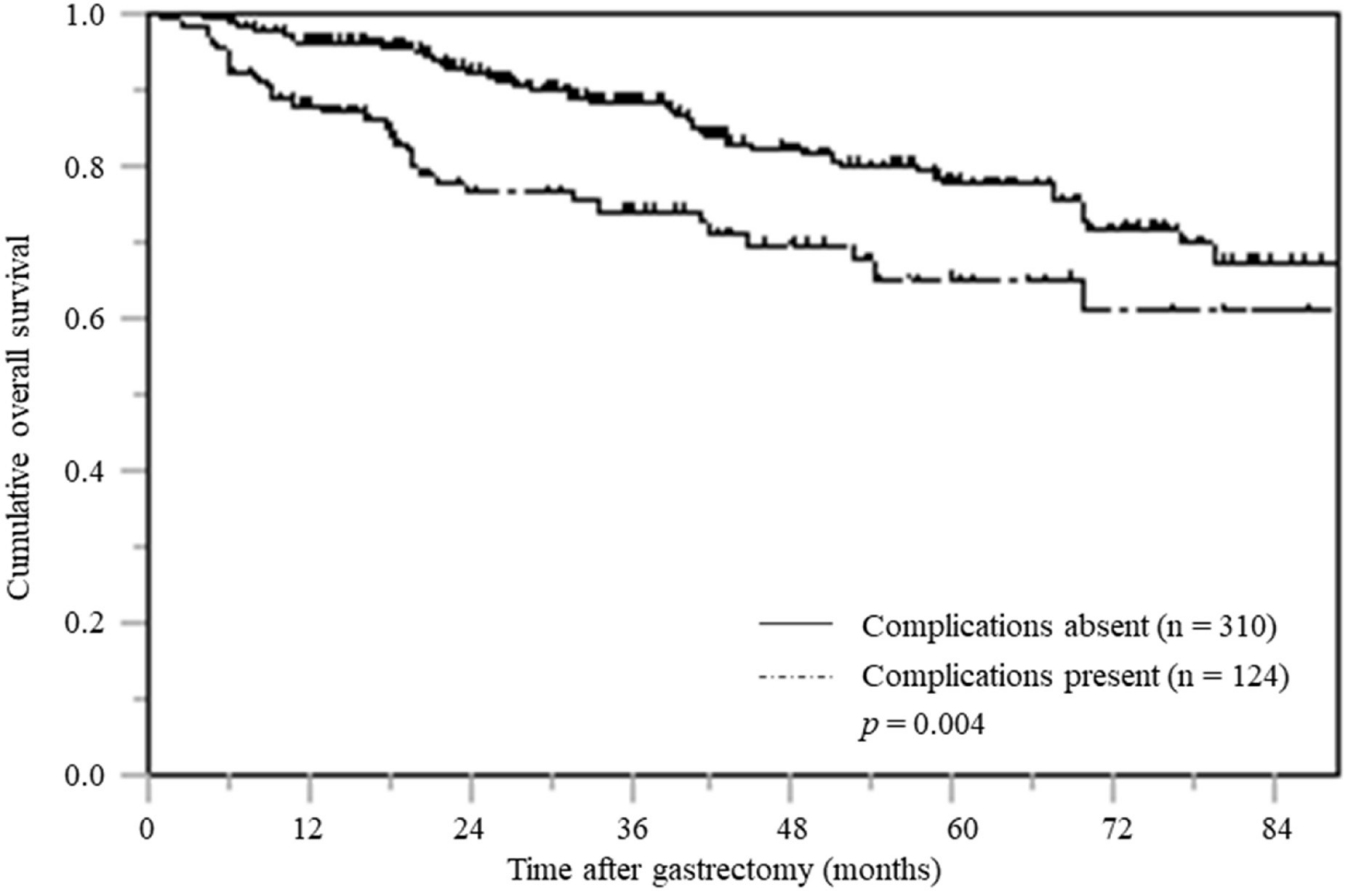
Overall survival based on postoperative complications (CD≧II).

The OS of the patients with postoperative complications was 74.2% and 65.2% at three-year and five-year, respectively. The OS of the patients without postoperative complications was 88.5% and 77.7% at three-year and five-year, respectively. The OS curve for the patients with postoperative complications was significantly inferior to that of the patients without complications (*p* = 0.004) (Figure 2).

### OS and complications stratified by PNI

**Figure 3:**
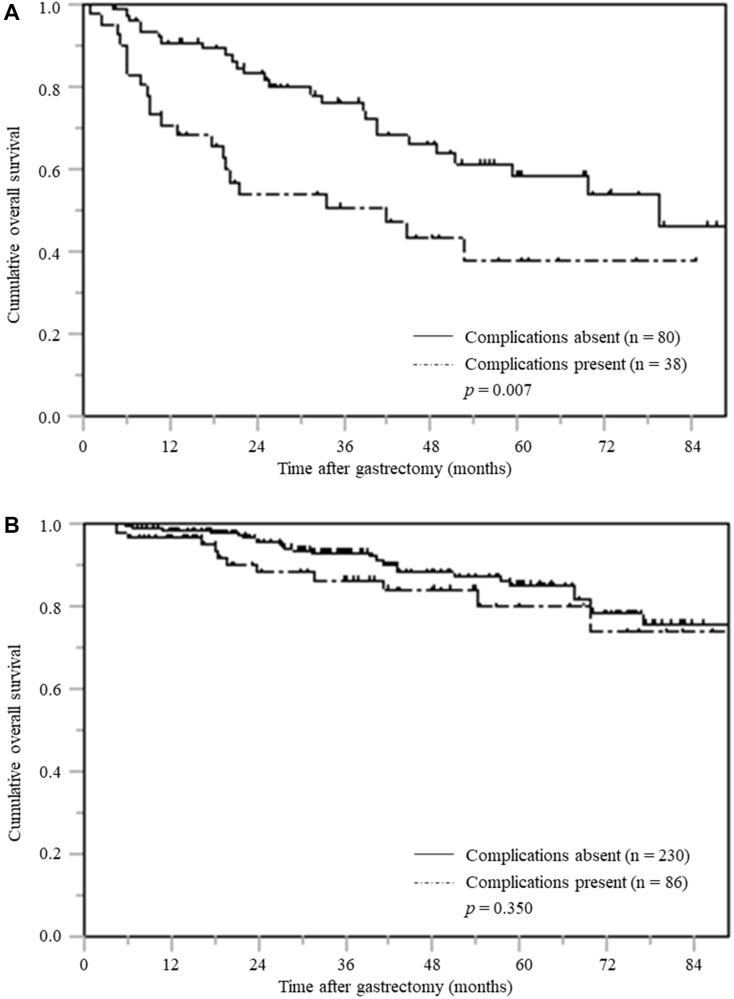
Overall survival based on postoperative complications stratified according to PNI. (**A**) Low PNI (**B**) High PNI.

Among the 118 patients with low PNI, the three-year and five-year OS in the patients with postoperative complications was 50.8% and 37.8%, respectively; while the three-year and five-year OS in the patients without complications was 76.2% and 58.4%, respectively. Among patients with low PNI, those with postoperative complications had significantly inferior OS than those without postoperative complications (*p* = 0.007) (Figure 3A).

However, in 316 patients with high PNI, there was no significant difference in OS between those with and without postoperative complications. (*p* = 0.350) (Figure 3B).

### OS based on postoperative complications stratified by pTNM stage

**Figure 4:**
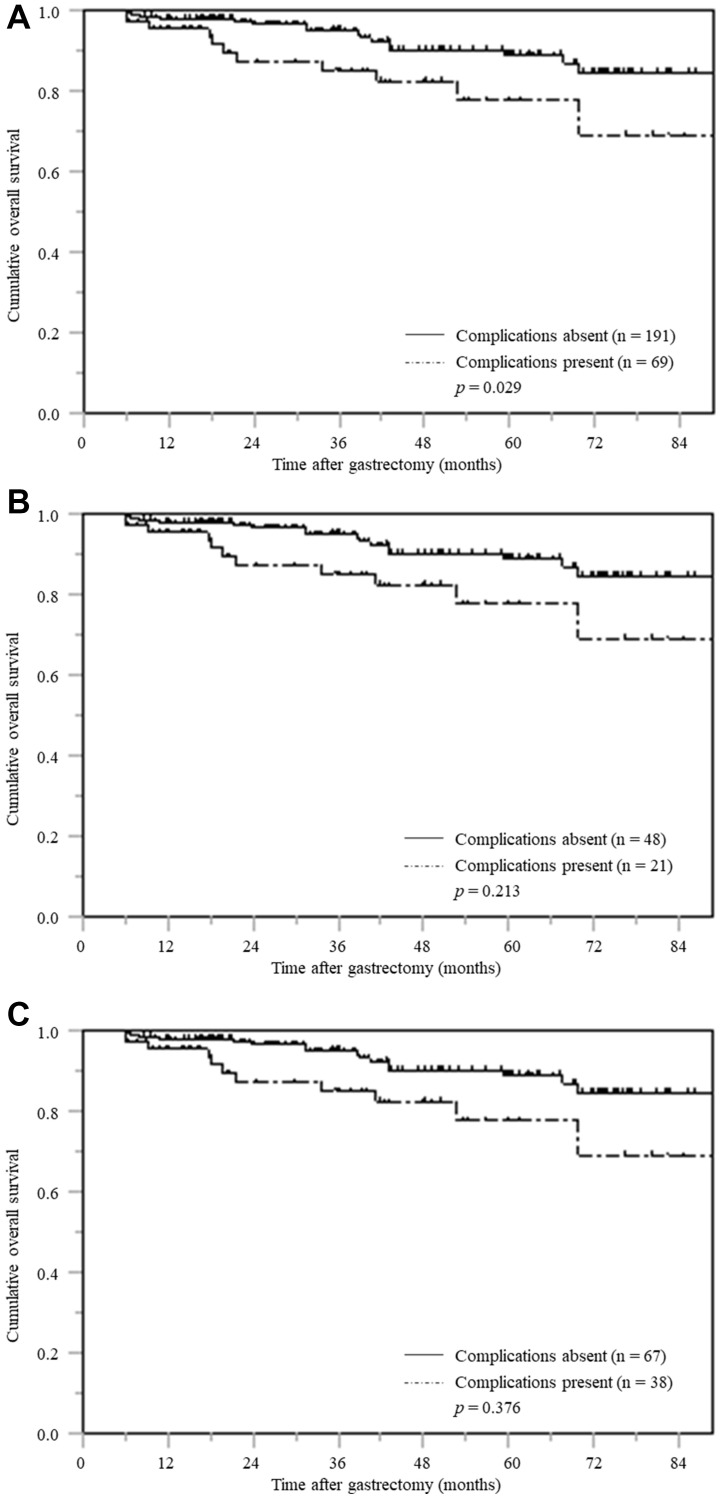
Overall survival based on postoperative complications stratified according by pTNM stage (**A**) pTNM stage I (**B**) pTNM stage II (**C**) pTNM stage III.

Of the 260 patients with pTNM stage I, the OS curve for the patients with postoperative complications was significantly inferior to that of the patients without postoperative complications (*p* = 0.029) (Figure 4A).

However, among patients with pTNM stages II and III, the OS of those with and without postoperative complications did not differ significantly (Figure 4B and 4C).

### OS based on postoperative complications stratified by PNI for each pTNM stage

**Figure 5:**
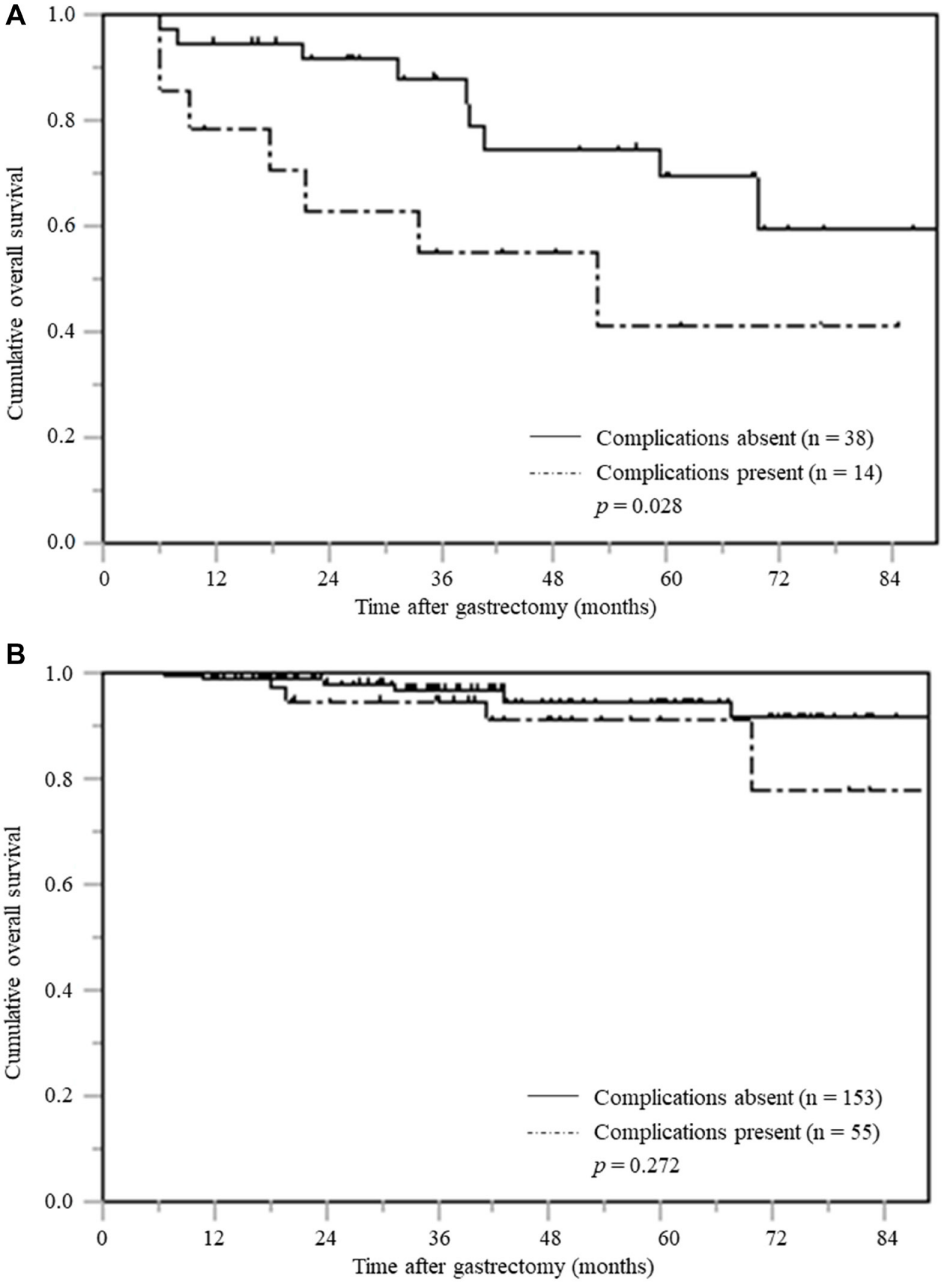
Overall survival based on postoperative complications stratified by PNI in patients with pTNM stage I. (**A**) Low PNI (**B**) High PNI.

**Figure 6:**
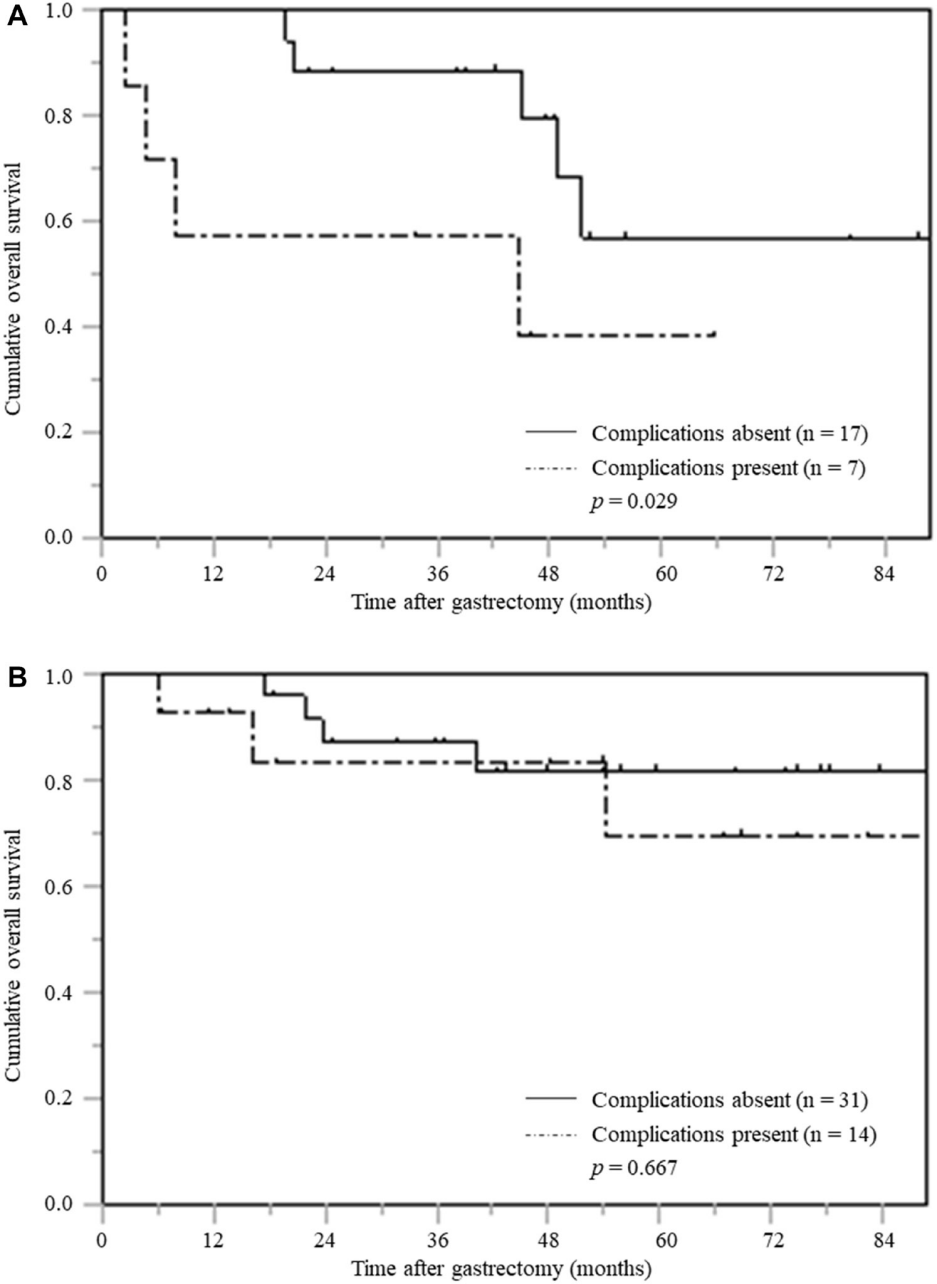
Overall survival based on postoperative complications stratified by PNI in patients with pTNM stage II. (**A**) Low PNI (**B**) High PNI.

Among patients pTNM stage I patients with low PNI, the OS curve for the patients with postoperative complications was significantly inferior to that of the patients without postoperative complications (*p* = 0.028) (Figure 5A). However, among high PNI patients, there was no significant difference in OS among those with and without postoperative complications (Figure 5B). Similarly, among pTNM stage II patients with low PNI, those with complications had significantly inferior OS than those without postoperative complications (*p* = 0.029) (Figure 6A). However, there was no significant difference in OS between patients with and without postoperative complications in high PNI group (*p* = 0.667) (Figure 6B).

**Figure 7:**
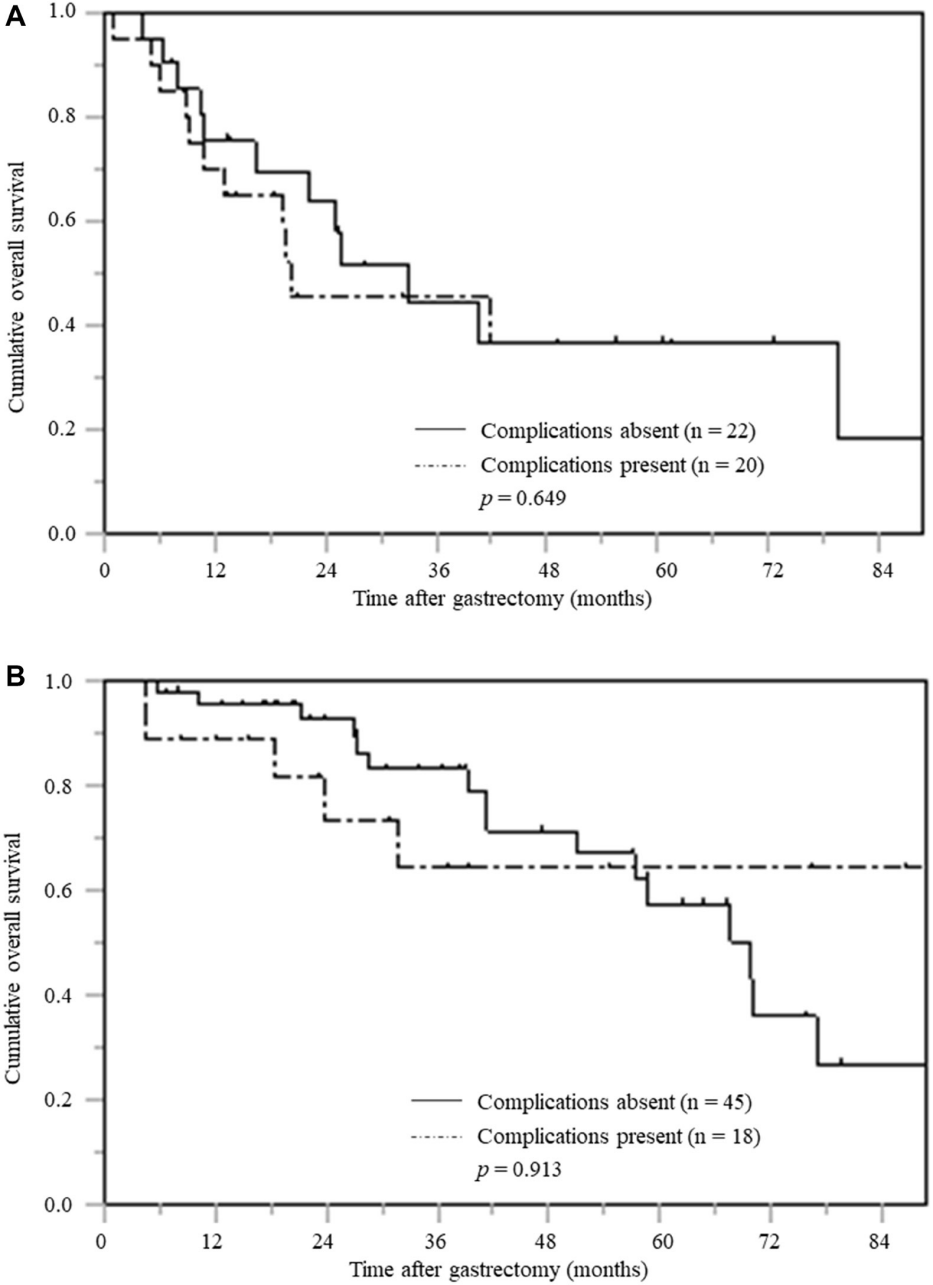
Overall survival based on postoperative complications stratified by PNI in patients with pTNM stage III. (**A**) Low PNI (**B**) High PNI.

Meanwhile, among pTNM stage III patients, there was no significant difference in OS between those with and without postoperative complications when stratified according to PNI value (Figure 7A and 7B).

## DISCUSSION

Recent studies have shown that postoperative complications such as anastomotic leakage and postoperative infections adversely affect the prognosis of gastric cancer patients [3, 17–20]. Similarly, in this study we confirmed that presence of postoperative complications significantly predicted worse prognosis, and the type of surgical procedure and PNI were independent risk factors for postoperative complications. Interestingly, long-term prognosis was found to unaffected by postoperative complications among well-nourished gastric cancer patients (i.e., patients with a high preoperative PNI). Although the degree of immunosuppression was not assessed in this study, we consider that postoperative complications increased surgical stress due to local and systemic inflammatory responses, resulting in more severe immunosuppression in the low PNI group than in the high PNI group [21, 22]. Therefore, well-nourished patients may be able to tolerate immunosuppression associated with inflammatory cytokines induced by postoperative complications [3, 23, 24]. Accordingly, we speculated that PNI can be a comprehensive indicator of nutrition and immunological status in gastric cancer patient. Preoperative nutritional interventions, based on preoperative PNI assessments, can reduce the postoperative complication and lead to improvement in therapeutic effect and long-term prognosis. However, because of the limited time between cancer identification and therapeutic surgery, preoperative malnutrition cannot be improved substantially in a short term for advanced cancer [25, 26]. The results of several studies have been inconsistent regarding the efficacy of preoperative nutritional intervention, and it may be difficult to introduce preoperative nutritional interventions to improve OS.

In the stage-stratified analysis, pTNM stage I patients who developed postoperative complications had a significantly inferior prognosis than those without complications, whereas there was no difference in prognosis with respect to postoperative complications in stage II and III patients. In addition, among stage I and II gastric cancer patients, those with low PNI who developed postoperative complications had a significantly inferior OS compared to those without postoperative complications, whereas there was no difference in high PNI patients. And among pTNM stage III patients, there was no significant difference in OS between those with and without complications, regardless of PNI value. These findings suggest that preoperative nutritional status, similar to postoperative complications, is crucial in the prognosis of gastric cancer, especially in relatively early-stage cancer, whereas the association of degree of cancer stage outweighs that of postoperative complications with OS in advanced-stage cancer [27, 28].

In clinical practice, TNM staging alone cannot be used to predict clinical outcomes, since it only classifies patients according to postoperative pathological outcomes and does not include nutritional or inflammatory status [29, 30]. Thus, novel relevant prognostic predictors are needed to improve prognosis with individual treatment for gastric cancer. Recently, researchers have focused not only on tumor itself, but also on the tumor’s microenvironment, especially the immunonutrition and inflammatory status [31–33]. This study showed that PNI is important in assessing the risk of postoperative complications and is an independent predictor of long-term prognosis in gastric cancer patients. Considering the fact, we believe that PNI can effectively complement TNM staging and provide valuable information for individualized prognosis of gastric cancer patients.

While the current study has some advantages over previous reports, it has some potential limitations and caution should be exercised when interpreting the results. First, this was a single-institutional study with a relatively small number of patients and a relatively short follow-up period for evaluation of long-term prognosis. The follow-up period was sufficient to assess the outcome of postoperative complications, but further research is needed to investigate the impact of PNI on long-term prognosis. Second, immunonutritional parameters and systemic levels of inflammatory cytokines were not assessed. Third, the subgroup-derived evidence did not have sufficient statistical power to validate various conclusions because of the small number of patients. Fourth, the analysis did not include factors that could affect inflammation and nutritional markers, such as medication and comorbidity. Fifth, OS was evaluated in this study as it is considered the gold standard endpoint in cancer prognosis studies. However, disease-specific survival and recurrence-free survival data analysis would also provide to be useful. They were not analyzed.

In conclusion, this study suggested that PNI is useful in identifying gastric cancer patients who would benefit from preoperative nutritional management and have an improved prognosis. However, it is unclear whether PNI serve as a nutritional parameter for selecting candidates for nutritional management. Future prospective studies are needed to determine whether aggressive preoperative nutritional management can increase preoperative PNI and improve short-term outcomes and long-term prognosis.

## MATERIALS AND METHODS

### Patients

We retrospectively investigated the medical records of 434 gastric cancer patients who undergoing curative laparoscopic gastrectomy between 2010 and 2018. The gastrectomy and lymphadenectomy were determined by the Japanese Gastric Cancer Treatment Guidelines (version 4) [34]. The pathological classification was judged based on the International Union Against Cancer (UICC) TNM 7th edition [35]. The ethics committee of Shimane university approved this study.

### Outcomes

Postoperative complications of grade II or higher according to the Clavien-Dindo (CD) classification were retrospectively determined from the patients’ records [36]. Postoperative complications associated with gastric resection were defined as bleeding, anastomotic leakage and stenosis, pancreatic fistula, ascites, surgical site infection, abscess, pleural effusion, deep vein thrombosis/ pulmonary embolism, intestinal paralysis and any organ disease or infection. OS was calculated from the date of gastrectomy to the date of death due to any cause or the last follow-up.

### Preoperative nutritional parameters

PNI and BMI, were calculated using laboratory data obtained within 1 week preoperatively. PNI was proposed by Onodera et al. [9].

**Figure 8:**
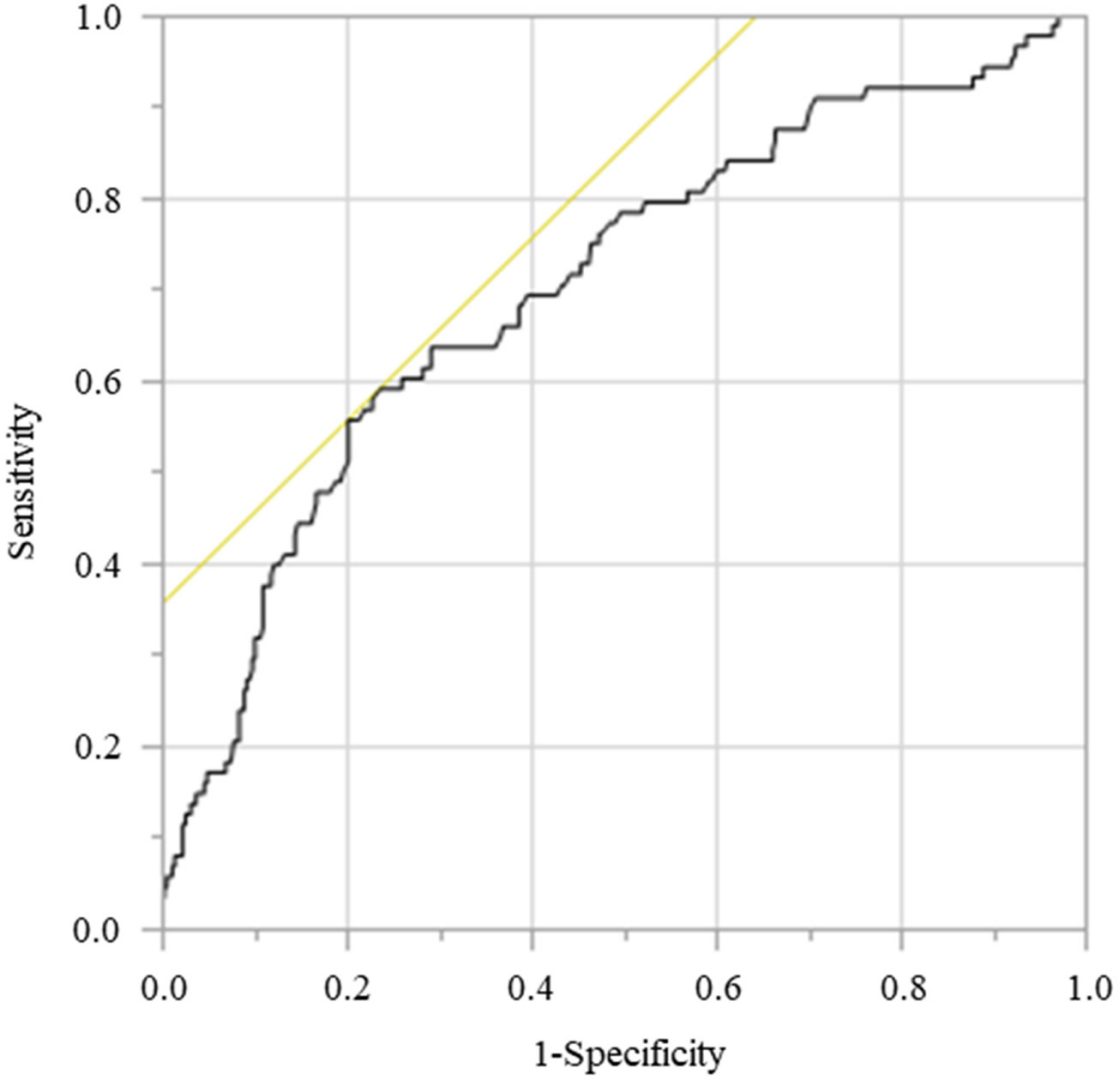
Receiver operating curve for OS was plotted to verify the optimum cut-off value of PNI.

The receiver operating characteristics (ROC) curve and area under curve (AUC) analyses were performed to determine the optimal cut-off value of PNI for OS. Based on this analysis, cut-off value of PNI was set at 44.2 (sensitivity, 55.7%; specificity, 79.8%; AUC = 0.701) (Figure 8).

### Statistical analyses

Continuous variables are represented as median and range. Differences between groups were assessed using Student’s *t*-test. Differences between categorical variables were analyzed using the chi-square test. The OS was analyzed using the Kaplan-Meier method and the Log-rank test. In addition, a univariate analysis was performed to identify significantly relevant variables, and variables with a univariate *p*-value <0.05 were included in the subsequent multivariate analysis. The Cox proportional hazards model used HR and a 95% confidence interval (95% CI). Statistical analysis was performed using JMP software (version 16, USA), and *p* < 0.05 was judged to be a statistically significant difference.

## Abbreviations

AUC: area under the curve
BMI: body mass index
CEA: carcinoembryonic antigen
CI: confidence interval
CD: Clavien-Dindo
CRP: C-reactive protein
HR: hazard ratio
OS: overall survival
PNI: Prognostic nutritional index
pStage: pathological stage
ROC: Receiver operating characteristic
TNM: tumor, node, metastasis
