# Correction: Influence of nutrition on stage-stratified survival in gastric cancer patients with postoperative complications

**DOI:** 10.18632/oncotarget.28243

**Published:** 2022-10-08

**Authors:** Noriyuki Hirahara, Takeshi Matsubara, Shunsuke Kaji, Yuki Uchida, Ryoji Hyakudomi, Tetsu Yamamoto, Kiyoe Takai, Yohei Sasaki, Koki Kawakami, Yoshitsugu Tajima

**Affiliations:** ^1^Department of Digestive and General Surgery, Shimane University Faculty of Medicine, Izumo, Shimane, Japan; ^2^Department of Surgery, Matsue Red Cross Hospital, Shimane, Matsue, Horomachi, Japan; ^3^Department of Surgery, Masuda Red Cross Hospital, Shimane, Masuda, Otoyoshi-cho, Japan

**This article has been corrected:** Figure 4 contains accidental duplicate images of panel A across panels B and C. The corrected Figure 4, produced using the original data, is shown below. The authors declare that these corrections do not change the results or conclusions of this paper.

Original article: Oncotarget. 2022; 13:183–197. 183-197. https://doi.org/10.18632/oncotarget.28179

**Figure 4:**
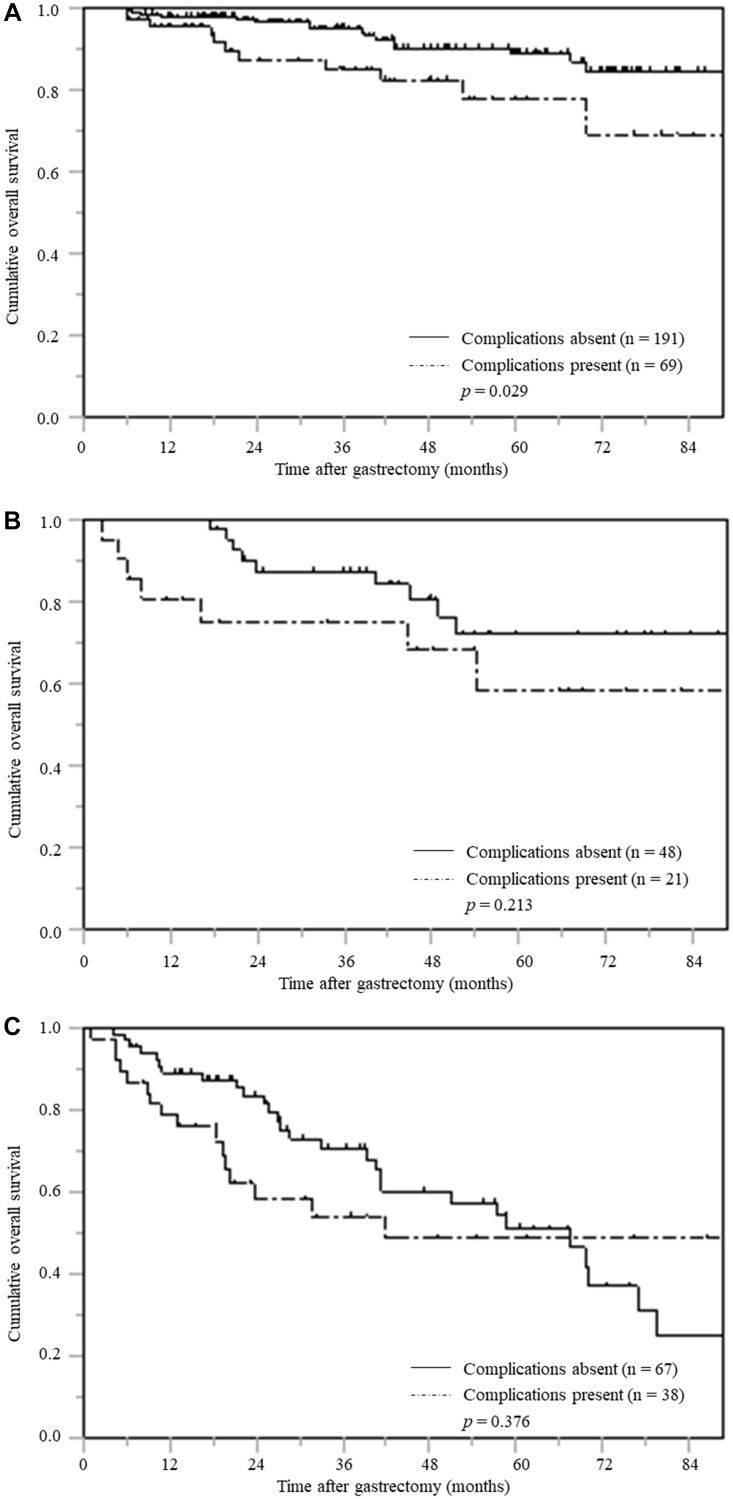
Overall survival based on postoperative complications stratified according by pTNM stage (**A**) pTNM stage I (**B**) pTNM stage II (**C**) pTNM stage III.

